# Endoscopic drainage and necrosectomy for inoperable gangrenous cholecystitis

**DOI:** 10.1055/a-2006-4369

**Published:** 2023-02-28

**Authors:** Beatrice Orlandini, Freek Daams, Paul Fockens, Rogier P. Voermans, Roy L. J. van Wanrooij

**Affiliations:** 1 Unit of Clinical Gastroenterology, Careggi University Hospital, Florence, Italy; 2 Department of Surgery, Cancer Center Amsterdam, Amsterdam UMC, Vrije Universiteit Amsterdam, the Netherlands; 3 Department of Gastroenterology and Hepatology, Amsterdam Gastroenterology Endocrinology Metabolism, Amsterdam UMC, University of Amsterdam, Amsterdam, the Netherlands; 4 Department of Gastroenterology and Hepatology, Amsterdam Gastroenterology Endocrinology Metabolism, Amsterdam UMC, Vrije Universiteit Amsterdam, the Netherlands

A 56-year-old man with liver, peritoneal, and lymph node metastases after previous transthoracic esophagectomy was diagnosed with acute calculous cholecystitis. Transduodenal endoscopic ultrasound-guided gallbladder drainage (EUS-GBD) was performed using a 10 × 10 mm cautery-enhanced lumen-apposing metal stent (LAMS). Purulent fluid was aspirated and submitted for culture, which identified *Enterococcus faecium.*

After EUS-GBD the patient initially recovered well, but 3 weeks later fever and abdominal pain recurred. A computed tomography (CT) scan revealed a persistent hydropic gallbladder suggesting stent dysfunction. An upper endoscopy was then performed, which showed obstruction of the LAMS with necrotic tissue ( Fig. 1 ). Using forceps and a snare, the necrotic tissue was removed from the stent and gallbladder ( Video 1 ). The largest specimen removed measured 9 × 1.5 cm and histopathological examination revealed a necrotic gallbladder wall ( Fig. 2 ). Complete evacuation of debris was confirmed by cholecystoscopy, which also showed a vital gallbladder wall. Two double-pigtail stents, 7 Fr × 5 cm, were placed to allow long-term drainage. Hereafter the patient showed significant clinical improvement without recurrence of fever or pain. A CT scan performed 2 weeks later revealed a collapsed gallbladder without signs of inflammation.

**Fig. 1:**
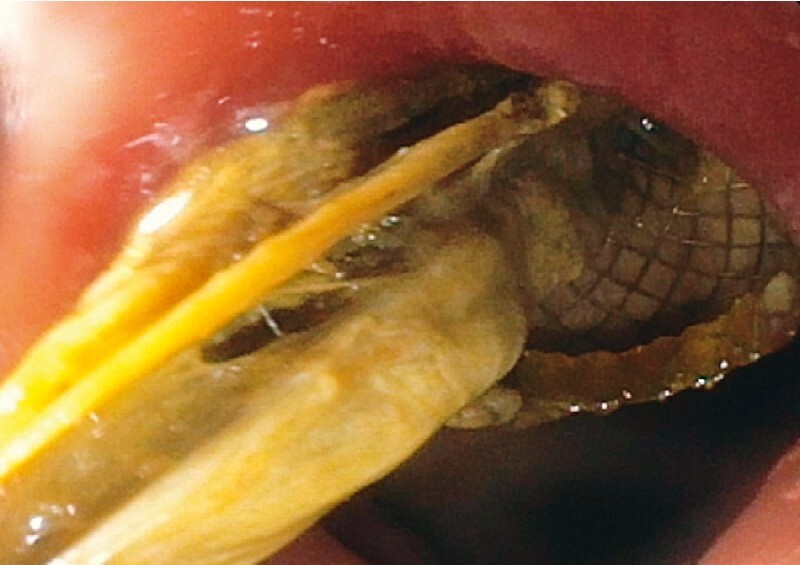
Endoscopic image of lumen-apposing metal stent obstructed with necrotic tissue.

**Video 1** Endoscopic removal of necrotic gallbladder tissue through a lumen-apposing metal stent.


**Fig. 2:**
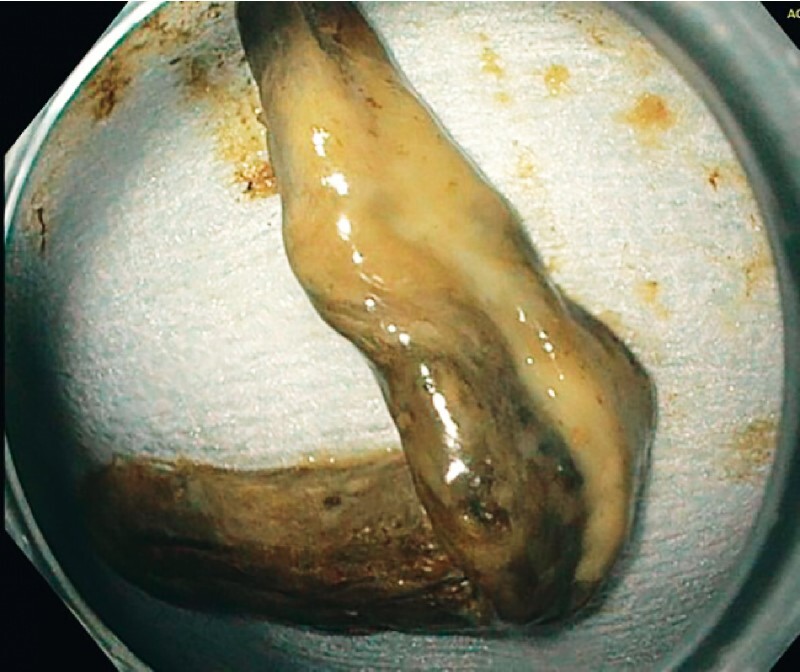
The largest specimen removed (9 × 1.5 cm) was sent for histopathological examination, which confirmed a necrotic gallbladder wall.

EUS-GBD using LAMS is an innovative technique for patients with acute cholecystitis who are unfit for surgery 1 . EUS-GBD is preferred over percutaneous drainage as it allows internal drainage and provides access to the gallbladder, which facilitates stone removal 2 . In this case, acute cholecystitis was complicated by gangrenous cholecystitis, which is a severe form of acute cholecystitis characterized by ischemia and necrosis of the gallbladder wall 3 . The solid necrotic tissue in the gallbladder contained pockets of pus, which impeded adequate drainage. The LAMS allowed complete removal of the necrotic tissue by endoscopy.

In conclusion, to the best of our knowledge, we present the first report of endoscopic drainage and subsequent endoscopic necrosectomy of gangrenous cholecystitis.

Endoscopy_UCTN_Code_TTT_1AS_2AD

## Citation Format

Endoscopy 2023; 54 (S01); E112-E113, DOI: 10.1055/a-1948-2124.

## Notes

Endoscopy E-Videos

https://eref.thieme.de/e-videos
*Endoscopy E-Videos* is an open access online section, reporting on interesting cases and new techniques in gastroenterological endoscopy. All papers include a high quality video and all contributions are freely accessible online. Processing charges apply (currently EUR 375), discounts and wavers acc. to HINARI are available. This section has its own submission website at https://mc.manuscriptcentral.com/e-videos
